# Prevalence and Associated Risk Factors of Bovine Tuberculosis in Dairy Cattle Determined by Comparative Intradermal Tuberculin Test in Mali and Niger, 2024

**DOI:** 10.3390/pathogens15040421

**Published:** 2026-04-14

**Authors:** Abel Biguezoton, Haladou Gagara, Chaka Traore, Der Dabire, Zakaria Bengaly, Mahaman Maaouia Abdou Moussa, Kader Issoufou, Maïmouna Ousmane, Marcella Mori, Claude Saegerman

**Affiliations:** 1 Vector-Borne Diseases and Biodiversity Unit (UMaVeB), International Research and Development Centre on Livestock in Sub-Humid Areas (CIRDES), Bobo-Dioulasso BP 454, Burkina Faso; dsdabire@yahoo.fr (D.D.); zakaria.bengaly@cirdes.org (Z.B.); 2 Laboratoire Central de l’élevage (LABOCEL), Niamey BP 485, Niger; haladoug@yahoo.fr; 3 Laboratoire Central Véterinaire (LCV), Bamako BP 2295, Mali; traorechaka2000@yahoo.fr; 4 Faculté d’Agronomie (FA), Université Abdou Moumouni (UAM), Niamey BP 10896, Niger; maaouia_abdou@yahoo.fr; 5 Belgian Agency for International Cooperation (Enabel), Niamey BP 12987, Niger; kaderiss2006@yahoo.fr (K.I.); maimounaousmane67@gmail.com (M.O.); 6 Sciensano, Belgian Institute for Health, 1050 Brussels, Belgium; marcella.mori@sciensano.be; 7 Research Unit in Epidemiology, Risk Analysis and Biosecurity Applied to Veterinary Sciences (UREAR-ULiege), Fundamental and Applied Research for Animals & Health (FARAH) Center, Faculty of Veterinary Medicine, University of Liege, 4000 Liege, Belgium

**Keywords:** cattle, dairy, zoonosis, tuberculosis, prevalence, risk factor, classification tree analysis, biosecurity

## Abstract

Background: Bovine tuberculosis (bTB) caused by *Mycobacterium bovis* is a major zoonotic disease in West Africa. In Africa, bTB is endemic in cattle with a prevalence ranging from 2% up to 18%. The disease causes significant public health risks due to unpasteurized milk and milk product consumption. In the context of the EU-PRISMA project, which promotes research and innovation for productive, resilient, and healthy agropastoral systems in West Africa, a cross-sectional survey was conducted in dairy herds from Mali and Niger to assess animal, herd, and within-herd bTB prevalence, as well as to identify animal risk factors and predictors of bTB herd status. Method and principal findings: A random cross-sectional survey on dairy cattle farms using comparative intradermal tuberculin test and epidemiological inquiry was performed in four regions of Mali (Bamako, Koulikoro, Mopti, and Sikasso) and three regions of Niger (Tahoua, Dosso, and Tillabéry). Herd and animal prevalence of bTB and within-herd prevalence were significantly higher in Mali (especially in Bamako and Koulikoro) than in Niger. Several risk factors were significantly associated with animals positive to bTB, i.e., the region where animals live, the age range from 3 to 7 years old, and female animals. In addition, in regions with higher bTB prevalence, the herd with slaughtering of animals in the farm and the herd with the presence of an animal assembly area were associated with the most unfavorable status of a herd with regards to bTB. Moreover, the average and the median annual economic losses of bTB at animal level were estimated at €262 and €137 respectively, with large variability depending on the farm (between €46 and €838). Conclusion and significance: This survey provides useful data on bTB epidemiology and economical losses in Mali and Niger and urges for improvement of surveillance systems and prevention and control strategies. Cost-benefit, return of investment, or similar analyses are strongly recommended to help with decision making.

## 1. Introduction

Bovine tuberculosis (bTB) caused by infection with *Mycobacterium bovis* (*M. bovis*) remains one of the most persistent and challenging veterinary and public health threats globally [1]. In sub-Saharan Africa—and specifically within the agropastoral belts of Mali and Niger—bTB remains a neglected endemic disease with profound implications for food security, animal welfare, and rural livelihoods [2]. It is a major zoonotic disease in West Africa, endemic in cattle with varied prevalence and with significant public health risks due to the consumption of unpasteurized milk and milk products [3]. The disease is often missed by standard diagnostics, requiring better surveillance, cross-border control, and farmer biosecurity education for effective management.

The livestock sector is a foundational pillar of the West African economy. In Mali and Niger, both members of the West African Economic and Monetary Union (UEMOA), cattle production contributes significantly to the national gross domestic product (GDP), often exceeding 10% to 15% [4]. In recent years, both nations have shifted toward the intensification of dairy production to meet the rising nutritional demands of rapidly urbanizing populations. This transition from traditional, extensive transhumant systems to semi-intensive dairy farming has altered the epidemiological landscape. Higher animal density, shared water points, and the introduction of high-yielding exotic breeds—which may possess higher susceptibility compared to hardy local Zebu—have created an environment conducive to the maintenance and transmission of *M. bovis* [5,6]).

Despite these risks, bTB surveillance in Mali and Niger has historically been hampered by limited diagnostic infrastructure and the logistical complexities of tracking mobile herds. Accurate prevalence data is scarce, often relying on outdated slaughterhouse reports which frequently underestimate the true burden of the disease in live populations [7]. To address this, the comparative intradermal tuberculin test (CITT) remains the international gold standard for field screening. By measuring the delayed-type hypersensitivity (DTH) response to both bovine and avian purified protein derivatives (PPD), the CITT allows for the differentiation of *M. bovis* infection from exposure to non-tuberculous mycobacteria (NTM), a common confounding factor in tropical environments [5]. It is noteworthy that according to a recent meta-analysis, bTB prevalence in cattle was estimated at 5%, with a higher burden in West Africa, particularly on farms compared to slaughterhouses [8].

Based on the same meta-analysis, *M. bovis* prevalence in humans in Africa averages 0.75% in general. However, in West and East Africa the average can be double, increasing the prevalence in livestock workers and in drug-resistant human cases [8], emphasizing the pivotal role of prevention strategies (i.e., farm biosecurity and WASH—Water, Sanitation, and Hygiene) to protect human health. Although identification of *M. bovis* from cattle and human hosts in Mali has been reported [9], other agents from the mycobacterium tuberculosis complex also affect humans, such as *M. tuberculosis*, *M. caprae*, or even *M. orygis*, reinforcing the implementation of the One Health perspective to the disease.

Few surveys exist on bTB in Mali and Niger, despite the existence of epidemiosurveillance networks for priority animal diseases (EPIVET—Mali and RESEPI—Niger). The prevalence of bTB in Niger was estimated in 2013 at 3.6%, with a 95% confidence interval between 1.9 and 5.9% [10]. In Niger, several identifications of *M. bovis* from cattle were reported [10] and suspected in human [11]. Meanwhile, Mali’s herd prevalence was reported at 94.44% in 2002–2003, while at the individual level it was 18.6% [2].

As comprehensive epidemiological data on bTB is scarce in the sub-region, it is necessary to provide the authorities with up-to-date prevalence data and reported protective factors to limit the risks associated with bTB integrating a One Health perspective.

The current survey is part of the EU-supported PRISMA project, that has been launched to promote research and innovation for productive, resilient, and healthy agropastoral systems in West Africa [12]. The aims of this survey were to update the estimation of the bTB prevalence at herd, individual levels, and within-herd prevalence, to identify the risk and protective factors of bTB, to predict the status of the herd based on several characteristics of the herd and the farmer’s family, and to estimate animal economic losses due to bTB in Mali and Niger. This update will serve as a prerequisite to adapt and improve the current bTB preventive and control program.

## 2. Materials and Methods

### 2.1. Study Area and Sampling Design

Agropastoralism in Mali and Niger is a dynamic, multi-functional production system that integrates sedentary crop cultivation with mobile livestock rearing. It serves as the primary livelihood for the majority of rural populations in the West African Sahel, functioning as a sophisticated strategy to mitigate the risks associated with extreme climatic variability and nutrient-poor soils [13].

The core of this system is a functional synergy between agriculture and animal husbandry. Agropastoralists typically cultivate drought-tolerant cereals, specifically pearl millet (*Pennisetum glaucum*) and sorghum. These cereals provide “crop residues” (stover), which have become increasingly critical as natural pastures diminish. In the Malian Sahel, crop residues can constitute up to 45% of the total forage intake during the dry season [14]. In return, livestock provide organic manure. This “manure-mediated” nutriment transfer is essential for maintaining soil phosphorus and nitrogen levels, which are naturally deficient in the sandy soils of Niger’s southern arable fringe [15].

The distribution of agropastoralism is governed by the 300 mm to 600 mm isohyets. In Mali, the system is most robust in the Sudano-Sahelian zone (Ségou, Koulikoro, and Kayes). A defining feature is the Inner Niger Delta, where agropastoralists synchronize their movements with the seasonal recession of floodwaters to access high-protein *Oryza longistaminata* (red rice) and *Echinochloa stagnina* (bourgou) pastures [16]. In Niger, agropastoralism is confined to the southern strip (Maradi, Zinder, and Tillabéri). Due to the higher risk of total crop failure in Niger compared to Mali, livestock often serve as a “living bank,” sold during the lean season to purchase grain [17].

Nowadays, the traditional equilibrium of these systems is under pressure. Shorter rainy seasons have forced a shift toward early-maturing crop varieties and the adoption of climate-smart agriculture (CSA) to ensure food security [13]. In the Liptako-Gourma region (the tri-border area of Mali, Niger, and Burkina Faso), rising insecurity has curtailed traditional transhumance (seasonal mobility). This has led to “forced sedentarization,” causing localized overgrazing and increased competition for water points [18].

Mali possesses one of the largest livestock herds in West Africa. Cattle (14 million heads) account for approximately 11% of Mali’s total livestock, which includes over 115 million heads across all species. The highest density is found in the Mopti region, which serves as a major hub. Niger has a massive cattle population relative to its human population, making it a critical player in the regional meat and live-animal trade. While the north is arid, the cattle population in Niger is densely packed along the southern border with Nigeria. The herd is currently estimated at over 20.4 million heads. The country is known for its milking cows (estimated at nearly 4 million), primarily managed in agropastoral systems.

**Figure 1:**
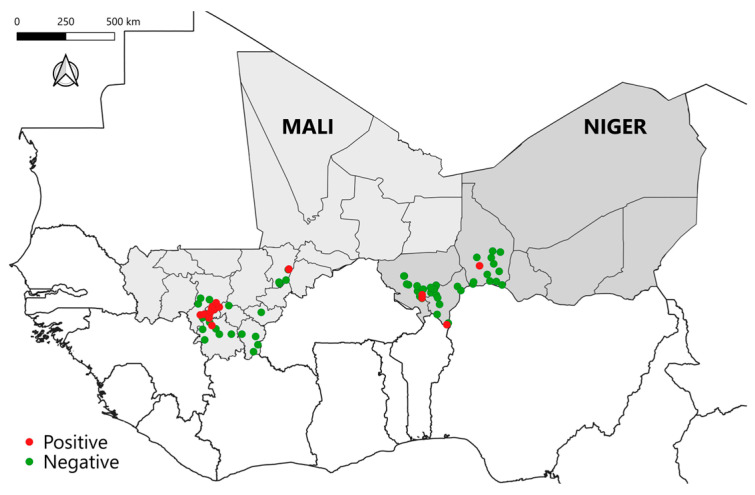
Geographic distribution of herds included in the bovine tuberculosis cross-sectional survey in Mali and Niger. Green and red dots represent seronegative and seropositive herds.

The research focused on dairy cattle farms in four regions of Mali (Bamako, Koulikoro, Mopti, and Sikasso) covering 43 locations, and in three regions of Niger (Tahoua, Dosso, and Tillabéry) covering 39 locations (Figure 1). Regions were selected based on levels of milk consumption and their inclusion within the main dairy production basins of each country. Within each region, sampling locations were randomly selected to ensure broad spatial coverage while taking accessibility and security constraints into account. Within each selected location (village), one herd was randomly chosen, and 15 animals were randomly sampled per herd. Indeed, each location corresponds to a herd. The survey unit (herd) was defined as a group of sedentary lactating cows managed together, owned by the same household, and kept in the same location.

The sample size was calculated based on the estimated number of lactating females in each country and region (Table 1), with an expected bTB prevalence (the proportion of a specific cattle population that is infected with *M. bovis* at a particular point in time) derived from previous studies (5%) [10], an accepted error of 1.25%, and a confidence level of 95%. The overall survey methodology was proposed by a team of epidemiologists and was collegially approved during methodological workshops held in both countries (year 2022), in which leading representatives from the human, veterinary, epidemiological, and laboratory sectors participated, representing a multidisciplinary One Health team.

### 2.2. Comparative Intradermal Tuberculin Test

The comparative intradermal tuberculin test (CITT) is used to differentiate between animals infected with *M. bovis* and those responding to bovine tuberculin as a result of exposure to other mycobacteria. This sensitization can be attributed to the antigenic cross-reactivity among mycobacterial species and related genera. The test involves the intradermal injection of bovine tuberculin and avian tuberculin (purified protein derivatives) [19].

**Figure 2:**
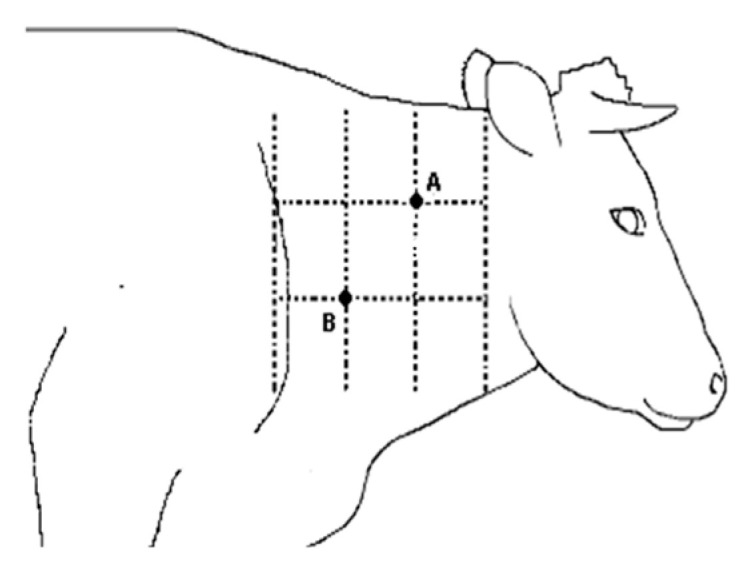
Elective area of the skin used for CITT. Legend: **A**, avian tuberculin; **B**, bovine tuberculin.

The CITT was performed according to the WOAH recommendation [19]. Briefly, the injection sites were clipped and cleaned. A fold of skin within each clipped area was measured with calipers and the site marked prior to injection. A short needle, with bevel edge facing outwards and attached to a graduated syringe filled with tuberculin, was inserted obliquely into the deeper layers of the skin (Figure 2).

The dose of tuberculin was then injected, and a single-dose syringe was used. A correct injection was confirmed by palpating a small pea-like swelling at each injection site. The distance between the two injections was approximately 12–15 cm. The skin-fold thickness of each injection site was re-measured 72 h after injection. The same person measured both the skin before the injection and at the time of reading the test. In addition, before the field survey, theoretical (April–May 2023) and practical (May 2024) workshops were organized with the technicians involved in the CITT to guarantee the technical procedure in the field.

The sensitivity and the specificity of the CITT was previously reported as 80% and 99.98% respectively [20,21].

### 2.3. Determination of Prevalence

The prevalence of bTB at animal and herd levels was calculated as the proportion of positive samples among the total number tested, and the 95% confidence interval was calculated using an exact binomial distribution [22]. An animal was considered positive if the increase in skin thickness at the bovine site of injection was 4 mm greater than the reaction shown at the site of the avian injection. The reaction was considered inconclusive if the increase was less than 4 mm and negative if the increase was less or equal to that of the avian site of infection [19].

### 2.4. Epidemiological Inquiry

An epidemiological survey was conducted to investigate animal characteristics and clinical signs, farmers’ livestock management practices, and human behavior and symptomatology. The survey aimed to identify potential risk factors or predictors for disease transmission. The data collected are summarized in Table A1.

### 2.5. Statistical Analyses and Cartography

Analyses were conducted using STATA SE 14.2 (StataCorp, College Station, TX, USA). The comparison of skin thickness between elective sites for avian and bovine tuberculin before injection was tested using the two-sample Wilcoxon rank-sum test. The 95% confidence interval (CI) of bTB prevalence was estimated using the exact binomial distribution. Comparison of bTB prevalence between countries or regions was tested using Fisher exact test. Risk and protective factors are factors that increase or decrease the likelihood of positive CITT and were identified using univariate and multivariate logistic regressions analysis. A risk or a protective factor is claimed when the odds ratio is respectively more or less than 1, and if 1 is not included within its 95% CI. The model was progressively simplified by removing the least significant variable with a *p* > 0.05. The model was considered complete, either when all variables had a significant *p*-value (<0.05) or when it could not be further simplified without having a significant difference between the most complex and the simpler model (likelihood ratio test with *p* < 0.05). Any probability value (*p*-value) below 0.05 indicated a statistically significant difference [22].

Classification tree analysis (CTA) was then applied to identify explanatory variables influencing herd bTB status directly or indirectly and considering the bTB herd positive if at least one animal presented a positive CITT (Salford Predictive Modeler, Salford Systems, San Diego, CA, USA). A classification tree analysis (CART) is a discrimination method based on the construction of a binary decision tree. The goal is to construct subgroups of a population that are as homogeneous as possible for a given characteristic (for more details, see, e.g., ref. [23]).

Maps were generated using QGIS software (version 3.44) [24].

## 3. Results

### 3.1. Skin Thickness

**Figure 3:**
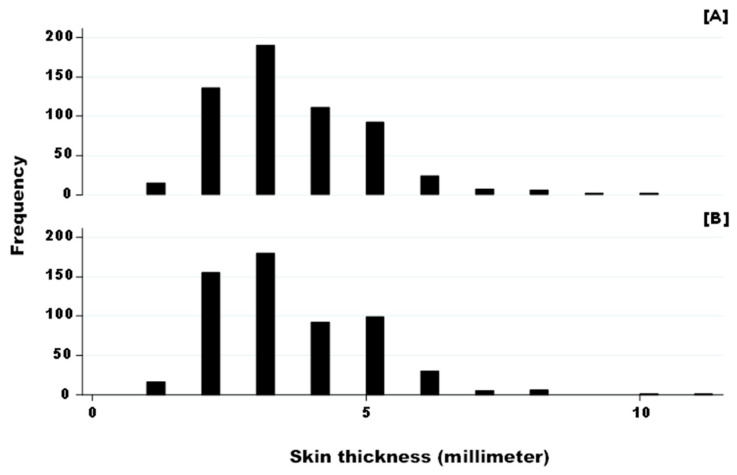
Skin thickness of animal from Niger (expressed in millimeter) before injection of avian (**A**) and bovine (**B**) tuberculin. Legend: Bars are individual animals with a certain skin thickness. Skin thickness average and standard error (SE) was 3.44 mm (SE: 1.44) and 3.48 mm (SE: 1.42) for (**A**) and (**B**), respectively.

This analysis was performed considering data from Niger (Figure 3). The skin thickness at T0 for both elective sites for avian and bovine tuberculin injection was similar (Two-sample Wilcoxon rank-sum test, z = 0.69 and *p*-value = 0.49).

### 3.2. Determination of Prevalence at Herd and Animal Levels

Overall, bTB herd prevalence was significantly higher in Mali with 44.19% (95% CI: 29.08–60.12) than in Niger with 10.25% (95% CI: 2.87–24.22) (Fisher exact test, *p*-value = 0.001) (Table 2).

**Figure 4:**
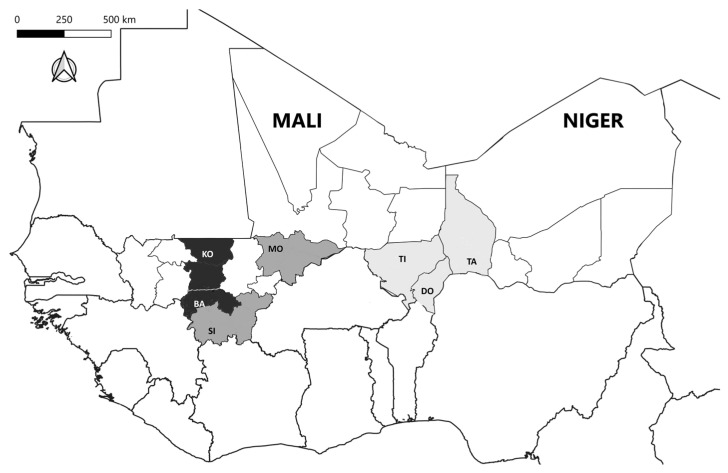
Risk map of animal bTB prevalence. Legend: BA, Bamako; DO, Dosso; KO, Koulikoro; MO, Mopti; SI, Sikasso; TA, Tahoua; TI, Tillaberi; light grey (animal prevalence < 1%); medium grey (animal prevalence between 1 and 5%); dark grey (animal prevalence between 10 and 15%).

At animal level, the bTB animal prevalence was significantly higher in Mali with 7.29% (95% CI: 5.40–9.57) than in Niger with 0.68% (95% CI: 0.19–1.74) (Fisher exact test, *p*-value < 0.001). Within Niger, no significant difference in prevalence was observed in either region. Unlike Mali, where Bamako’s (15.38% with 95% CI: 5.86–30.53) and Koulikoro’s (11.86% with 95% CI: 8.40–16.11) prevalence were higher than Mopti’s (3.96% with 95% CI: 1.09–9.83) and Sikasso’s (0.51% with 95% CI: 0.013–2.84) (Fisher exact test, *p*-value < 0.001), as depicted in Figure 4 where animal bTB prevalence clearly increases from east to west.

### 3.3. Within-Herd Prevalence

At country level, significantly higher within-herd prevalence was observed in Mali (Table 3) (Fisher exact test, *p* < 0.001). At region level, difference was observed only in Mali in relation with Bamako and Koulikoro, with significantly higher within-herd prevalence (Table 3) (Fisher exact test, *p* < 0.001).

### 3.4. Animal Risk Factors

Several risk (older animals and females) and protective (some regions from Mali like Mopti and Sikasso; and from Niger like Dosso, Tahoua, and Tillaberi) factors were hypothesized in this survey (Table 4).

### 3.5. Herd Predictors of Bovine Tuberculosis Status

A univariate logistic regression analysis was performed in order to select significant explanatory variables at herd and family levels that were related to the bTB status of the herd (see Table A1 for the details about these variables). After this, six variables were selected, i.e., four at herd level (region of the herd, presence of assembly areas, slaughtering of animals at the farm, and presence of animals that cry out (moan) repeatedly for no apparent reason), and two at family level (people suffering from persistent coughing and weight loss).

**Figure 5:**
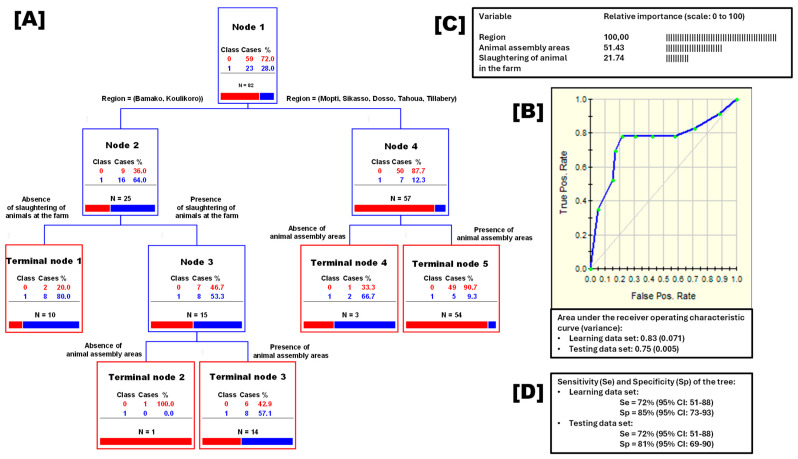
Classification tree identifying the main predictors of the bovine tuberculosis herd status. Legend: (**A**), the classification tree; (**B**), the associated ROC-curve; (**C**), the relative importance of the predictors, and (**D**) the sensitivity and the specificity of the classification tree for the learning and the testing data sets. Class: 0, for negative bovine tuberculosis herd status; Class 1, for positive bovine tuberculosis herd status. Cases: the number of herds concerned. %: percentage of herd concerned.

Later, a classification tree analysis (CTA) was done in order to select some predictors of the bTB status of a herd (Figure 5). The main predictors were the region of the herd (relative importance [RI] = 100; scale from 0 to 100), animal assembly areas (RI = 51.43), and slaughtering of animals in the farm (RI = 21.74). For both the learning data set (allowing the creation of the tree) and the testing data set (allowing to assess its predictability), the classification tree has an area under the receiver operating characteristic (ROC) curve of 0.75, a sensitivity of 72%, and a specificity above 80%.

## 4. Estimated Losses Due to Bovine Tuberculosis

**Figure 6:**
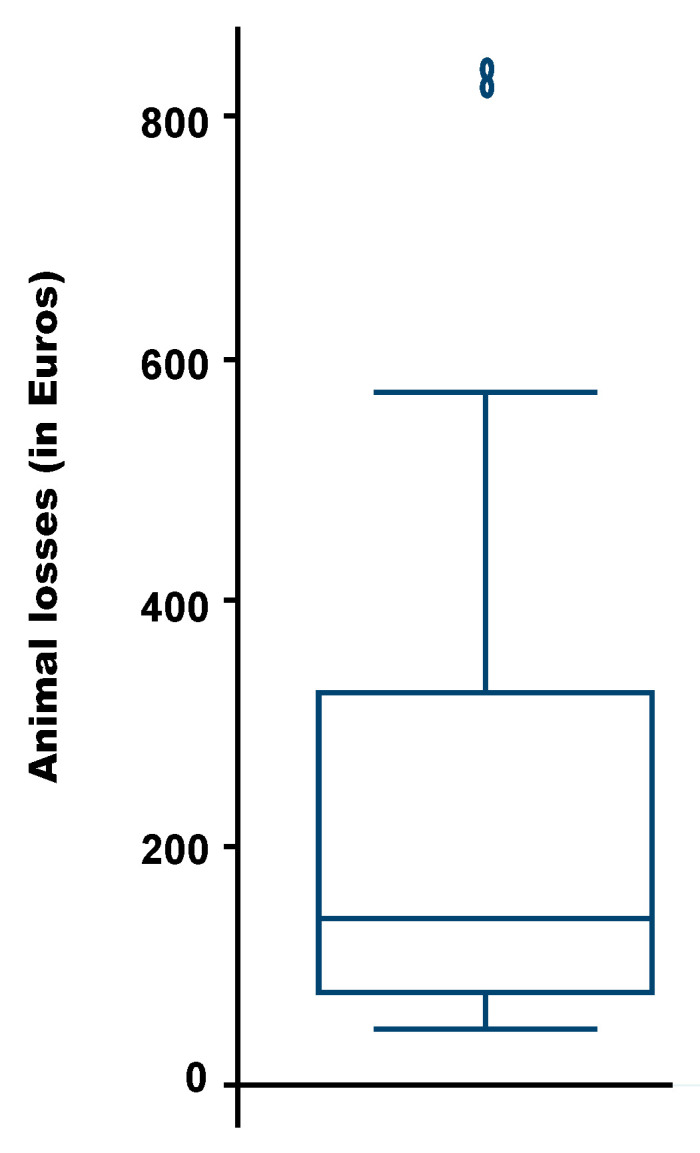
Box plot with the bTB annual losses estimated by the farmers in Mali and Niger.

The annual loss per animal due to bTB was estimated for 16 farms. The average loss was €262 (S.E.: €261; Min: €46; Max: €838) and the median loss of €137 (Figure 6).

## 5. Discussion

In Niger, the data on skin thickness before injection of avian and bovine tuberculin was statistically similar (3.48 mm and 3.44 mm in average) in both elective injection sites in the neck, despite some variability. It was hypothesized that this similarity allows for a good comparison of the CITT results. Similar average skin thickness was found in crossed breed dairy cattle in Bangladesh, another tropical area, with a value of 3.33 mm [25], confirming the plausibility of the measurements recorded in Niger. The variability is also supported by the literature as cattle skin thickness varies significantly by breed, age, sex, and environmental factors, generally ranging from thin (e.g., Zebu) to thick (e.g., Devon), with the breeds adapted to heat often having thinner skin [26].

Herd and animal prevalence of bTB is significantly higher in Mali (especially in Bamako and Koulikoro) than in Niger. In addition, at the country level, the within-herd prevalence was significantly higher in Mali in comparison with Niger. At the region level, the within-herd prevalence was significantly higher in Bamako and Koulikoro (>10% in 12/25 herds). According to a recent meta-analysis in Africa, the estimated animal bTB prevalence in cattle is 5.06% (95% CI: 3.76–6.78), with a higher burden in West Africa [8]. Indeed, animal bTB prevalence observed in this survey, with 7.29% in Mali, indicated a prevalence higher than the 95% CI of the previous meta-analysis. In addition, in the literature, the within-herd prevalence of bTB in dairy herds is highly variable with a median value of 10% [27]. The animal prevalence and the within-herd prevalence in a large number of herds tested from Bamako and Koulikoro are higher and call for the future development of prevention and control strategies.

The prevalence of bTB was significantly higher in Mali in comparison with Niger. With reference to Bamako, the prevalence of Koulikoro and Mopti was not significantly different but significantly less for other regions, indicating the need for an adaptation of the prevention and control program in first regions. In West Africa, bTB is known to be more prevalent than in the rest of Africa and varies depending on the country [8]. Identification of regions with a high prevalence suggests the necessity to target an awareness raising campaign in these regions. Another risk factor identified was the age of the animals, especially between 7 and 10 years. In Africa, according to ref. [28,29], the mean age of first calving is frequently 61.8 ± 12.85 months and the reproduction career has a mean age of 10.4 ± 3.1 years. Indeed, the probability of dairy cattle exposure increases with the age of the animals [30,31]. In addition, significantly more female bovines are found positive to CITT in comparison with male animals. A previous cross-sectional study in Uganda revealed significantly more females with positive skin tests compared to males [32]. However, in Africa, gender can be related to the age, given that dairy cows usually reach an older age due to their role in calving and milk production [32]. Concerning the type of breed, a significant effect was observed both in the univariate (*Bos taurus indicus* less at risk than crossed breed) and multivariate analysis (*Bos taurus indicus* more at risk than crossed breed), indicating a possible confounding factor not discovered (e.g., difference in animal density, lifespans, innate immunity, or metabolic rates) considering complex interactions that occur between hosts, pathogens, and the environment [32]. Because only three *Bos taurus taurus* animals were involved in this survey, a broader sample to clarify breed divergence is recommended. In the literature, few publications support that *Bos taurus indicus* is less at risk than *Bos taurus taurus* (e.g., [33,34]), but the difference in terms of susceptibility between breeds can be related also to differences in management; for example, imported dairy animals are generally kept under intensive conditions [35].

A good classification tree (with sensitivity and specificity above 75% and 80%, respectively) was found. In Mopti, Sikasso, Tahoua, and Tillabery regions which showed a lower bTB prevalence, the presence of animal assembly areas has few effects on the bTB status of the herd. However, in regions of Bamako and Koulikoro, both showing a higher bTB prevalence, herds with the slaughtering of animals in the farm and with the presence of an animal assembly were associated with the most unfavorable status of a herd with regard to bTB.

Identifying key predictors of bTB herd status—including regional hotspots, high-density assembly areas, and on-farm slaughtering practices—underscores an urgent need for enhanced biosecurity training and education for farmers, veterinary paraprofessionals (persons who are authorized by the Veterinary Statutory Body to carry out certain designated tasks in a territory), and private practitioners. Knowledge translation to co-production between partners is needed to select the most appropriate farm biosecurity measures, considering the local context, the acceptability, and the feasibility [35].

According to the farmers, the average and median annual economic losses of bTB at animal level ranged from €262 and €137, with a large variability depending on the farm (between €46 and €838). As few farmers were involved in this survey (extreme values have a strong effect on the average), the estimated average was higher than the mean estimated in Ethiopia, which is around €190 per animal [36]. The wide variations in estimates can be explained by the farming systems and the breeds of animals involved. Further studies to estimate the ratio between the cost of the disease and the cost of the surveillance and control bTB program are strongly recommended, as this information from other diseases has given successful results—e.g., for foot-and-mouth disease in Niger [37]. If the ratio is less than one, surveillance and control programs are more cost-effective than bearing the cost of the disease.

## 6. Conclusions

This survey provides updated data on bTB epidemiology in Mali and Niger. Overall, these findings can inform national authorities and stakeholders about the current status of the disease and support the strengthening of surveillance, prevention, and control strategies. In addition, awareness-raising campaigns should be initiated regarding bTB detection, the risks, and the protective factors. These campaigns should adopt a One Health perspective, targeting private veterinarians, veterinary paraprofessionals, farmers and their families, as well as medical doctors and healthcare personnel.

Future studies should consider sampling more regions, gathering larger sample sizes, and collecting samples suitable for the direct isolation of circulating *Mycobacterium* strains—targeting, for instance, carcasses seizures in the main slaughterhouses of both countries. This would enable the identification of the specific *Mycobacterium* species responsible for bTB in the selected areas and provide a more comprehensive understanding of the epidemiology and dynamics of bTB in Mali and Niger, thus updating and strengthening prevention and control programs to local realities.

## Figures and Tables

**Table 1:** Estimated number of cattle and lactating cows in the survey region of Mali and Niger.

Country	Region	Number of Cattle	Number of Lactating Cows
Mali	Koulikoro	1,617,000	291,192
	Sikasso	1,796,300	323,480
	Mopti	3,155,500	568,247
	Bamako	37,400	6735
	Sub-total	6,606,200	1,189,654
Niger	Dosso	1,984,437	264,921
	Tahoua	3,838,052	512,378
	Tillaberi	3,526,844	560,768
	Sub-total	9,349,333	1,338,067
	Total	15,955,533	2,527,721

**Table 2:** Bovine tuberculosis prevalence in Mali and Niger in the survey regions.

Country	Region	Herd Level			Animal Level		
		Positive (Prevalence in %)	Negative	Tested	Positive (Prevalence in %)	Negative	Tested
Mali	Bamako	3 (100)	0	3	6 (13.3)	39	45
	Koulikoro	13 (59.1)	9	22	35 (10.6)	295	330
	Mopti	2 (28.6)	5	7	4 (3.8)	101	105
	Sikasso	1 (9.1)	10	11	2 (1.2)	163	165
	Sub-total	19 (44.2)	24	43	47 (7.3)	598	645
Niger	Dosso	1 (12.5)	7	8	1 (0.8)	119	120
	Tahoua	1 (7.7)	12	13	1 (0.5)	194	195
	Tillaberi	2 (11.1)	16	18	2 (0.7)	268	270
	Sub-total	4 (10.3)	35	39	4 (0.7)	581	585
Total		23 (28.0)	63	82	51 (4.1)	1179	1230

**Table 3:** Number of herds with a specific bovine tuberculosis within-herd prevalence in Mali and Niger in the survey regions.

		Within-Herd Prevalence			
		<5%	[5–10%[	[10–20%[	≥20%
Mali	Bamako	0	1	1	1
	Koulikoro	9	3	2	8
	Mopti	5	0	2	0
	Sikasso	10	0	1	0
	Sub-total	24	4	6	9
Niger	Dosso	7	1	0	0
	Tahoua	12	1	0	0
	Tillaberi	16	2	0	0
	Sub-total	35	4	0	0
Total		59	8	6	9

**Table 4:** Risk and protective factors of bovine tuberculosis.

Variable	Modality	Univariate Logistic Regression			Multivariate Logistic Regression		
		Odds Ratio	95% CI	*p*-Value	Odds Ratio	95% CI	*p*-Value
Region	Bamako	Reference	-	-	Reference	-	-
	Koulikoro	0.77	0.30–1.95	0.58	0.85	0.32–2.29	0.75
	Mopti	0.26	0.069–0.96	0.04 *	0.29	0.07–1.16	0.08
	Sikasso	0.080	0.016–0.41	0.002 *	0.09	0.02–0.46	0.004 *
	Dosso	0.05	0.006–0.47	0.008 *	0.01	0.0008–0.15	0.001 *
	Tahoua	0.03	0.004–0.29	0.002 *	0.006	0.0004–0.075	<0.001 *
	Tillaberi	0.049	0.009–0.25	<0.001 *	0.009	0.0009–0.072	<0.001 *
Type of breed	Crossed	Reference	-	-	Reference	-	-
	*Bos taurus indicus*	0.21	0.09–0.47	<0.001 *	6.94	1.38–34.88	0.019 *
	*Bos taurus taurus*	Omitted #					
Age	[1–3 years[	Reference	-	-	Reference	-	-
	[3–7 years[	3.62	1.24–10.56	0.019 *	2.27	0.74–6.69	0.15
	[7–10 years[	4.71	1.57–14.07	0.006 *	3.05	0.93–10.02	0.066 °
	≥10 years	3.54	0.98–12.74	0.054	1.05	0.38–5.91	0.56
Sex	Male	Reference	-	-	Reference	-	-
	Female	2.31	1.11–4.79	0.025	2.22	0.98–4.97	0.054 °°

Legend: CI, confidence interval; #, due to the effective (i.e., only three animals); *, *p*-value less than 0.05; ° after backward stepwise (missing sex variable), this variable becomes significant with an OR = 4.72 (95% CI: 1.54–14.48) and *p*-value = 0.007; °° after backward stepwise (missing age variable), this variable becomes significant with an OR = 2.83 (95% CI: 1.34–5.97) and *p*-value = 0.007.

## Data Availability

The raw data supporting the conclusions of this article will be made available by the authors on request.

## Abbreviations

The following abbreviations are used in this manuscript:

bTB	Bovine tuberculosis
CI	Confidence interval
CITT	Comparative intradermal tuberculin test
CTA	Classification tree analysis
DTH	Delayed-type hypersensitivity
GDP	Gross domestic product
NTM	Non-tuberculous mycobacteria
PPD	Purified-protein derivatives
ROC	Receiver operating characteristic
T0	Time before injection of tuberculin
USA	United States of America
USD	United States Dollar

## Appendix A

**Table A1:** List of documented research data collected during the epidemiological survey. Animal, herd, and human-level variables collected through the questionnaire, with their definitions and possible responses.

Type	Level	Variable	Possible Responses
Animal	Animal	Sex	Male; Female
Animal	Animal	Age group	[1–3 years[; [3–7 years[; [7–10 years[; ≥10 years
Animal	Animal	Species	Bos taurus indicus, Bos taurus taurus, Bos taurus taurus X Bos taurus indicus (crossed)
Herd management	Herd	Presence of animal assembly areas	Yes; No
Herd management	Herd	Household sanitary conditions	1 = Good; 2 = Moderate; 3 = Poor
Herd management	Herd	Number of lactating females present in the herd	1 ≤ 10; 2 = 10–19; 3 = 20–29; 4 = 30–39
Herd management	Herd	Washing hands before milking	Yes; No
Herd management	Herd	Washing of equipments before milking	Yes; No
Herd management	Herd	Washing hands after milking	Yes; No
Herd management	Herd	Washing of equipments after milking	Yes; No
Herd management	Herd	Disposal of carcasses	1 = In the open environment; 0 = Other
Herd management	Herd	Slaughtering of animals at the farm	Yes; No
Herd management	Herd	Are there any animals in your herd that are currently coughing?	Yes; No
Herd management	Herd	Do you have animals that have died from persistent coughing?	Yes; No
Herd management	Herd	Are there animals in your herd that cry out (moan) repeatedly for no apparent reason?	Yes; No
Human	Family	Milk consumption	1 = Fresh unboiled milk; 0 = Other
Human	Family	Are there people in your circle (one or more) suffering from persistent coughing and weight loss?	Yes; No

## References

[1] WOAH (2026). Bovine Tuberculosis. General Disease Information Sheets. WOAH, Paris, France. https://www.woah.org/fileadmin/Home/eng/Media_Center/docs/pdf/Disease_cards/BOVINE-TB-EN.pdf.

[2] Sidibé S.S., Dicko N.A., Fané A., Doumbia R.M., Sidibé C.K., Kanté S., Mangané O., Konaté B., Koné A.Z., Maïga M.S. (2003). Tuberculose bovine au Mali:résultats d’une enquête épidémiologique dans les élevages laitiers de la zone périurbaine du district de Bamako. Rev. D’élevage Méd. Vét. Pays Trop..

[3] Collins A.B., Floyd S., Gordon S.V., More S.J. (2022). Prevalence of *Mycobacterium bovis* in milk on dairy cattle farms: An international systematic literature review and meta-analysis. Tuberculosis.

[4] World Bank (2023). Mali and Niger: Livestock Sector Analysis and National GDP Contributions.

[5] Ayalew S., Habtamu G., Melese F., Tessema B., Ashford R.T., Chothe S.K., Aseffa A., Wood J.L.N., Berg S., Mihret A. (2023). Zoonotic tuberculosis in a high bovine tuberculosis burden area of Ethiopia. Front. Public Health.

[6] Müller B., Dürr S., Alonso S., Hattendorf J., Cláudio J.M.L., Parsons S.D.C., van Helden P.D., Zinsstag J. (2013). Zoonotic *Mycobacterium bovis*-induced tuberculosis in humans. Emerg. Infect. Dis..

[7] Zinsstag J., Schelling E., Roth F., Bonfoh B., de Savigny D., Tanner M. (2006). Human benefits of animal interventions for zoonosis control. Emerg. Infect. Dis..

[8] Ngwira A., Manda S., Karimuribo E.D., Kimera S.I. (2025). Meta-analysis of the prevalence of tuberculosis in cattle and zoonotic tuberculosis in humans in sub-Saharan Africa. One Health Outlook.

[9] Diallo M., Diarra B., Sanogo M., Togo A.C., Somboro A.M., Diallo M.H., Traoré B., Maiga M., Koné Y., Tounkara K. (2016). Molecular identification of *Mycobacterium bovis* from cattle and human host in Mali: Expanded genetic diversity. BMC Vet. Res..

[10] Boukary A.R., Thys E., Rigouts L., Matthys F., Berkvens D., Mahamadou I., Yenikoye A., Saegerman C. (2012). Risk factors associated with bovine tuberculosis and molecular characterization of *Mycobacterium bovis* strains in urban settings in Niger. Transbound. Emerg. Dis..

[11] Grandjean-Lapierre S., Thiberville S.D., Fellag M., Eghazarian C., Bouzid F., Gavril C., Drancourt M. (2018). Recurrent bilateral *Mycobacterium bovis* necrotizing epididymitis: A case report. BMC Res. Notes.

[12] PRISMA Projet de Recherche et Innovation Pour Des Systèmes Agro-Pastoraux Productifs, Résilients et Sains en Afrique de L’Ouest. https://open.enabel.be/en/NER/2410/p/projet-de-recherche-et-innovation-pour-des-systemes-agro-pastoraux-productifs-resilients-et-sains-en-afrique-de-l-ouest.html.

[13] Assoumana B.T., Hiernaux P., Ickowicz A., Leroy J.L. (2024). Awareness and use of improved livestock feed technologies among agro-pastoral households in the West African Sahel. Trop. Anim. Health Prod..

[14] Diarra S.S., Traoré B. (2024). Synergies between Crop Residue Management and Livestock Productivity in Central Mali. Afr. J. Agric. Res..

[15] Hiernaux P., Mougin E., Diarra L., Soumaguel N., Lavenu F., Tracol Y., Diawara M. (2022). Long-term Trends in Sahelian Agropastoral Systems: Productivity and Carbon Sequestration. Glob. Change Biol..

[16] Ickowicz A., Ancey V., Corniaux C., Duteurtre G., Poccard-Chappuis R., Manzano P., Krätli S., Ruiz Mirazo J. (2021). The Future of Pastoralism in the Sahel.

[17] Veldhuizen A.J., Gijsbers G., Slingerland M. (2024). Pastoralism in the Sahel: Contexts, Complexities and Curatives: A Case Study from Niger.

[18] OECD/SWAC (2025). West African Papers No. 42: Land Tenure and Transhumance in the Liptako-Gourma Region.

[19] OIE (2018). Chapter 3.4.6. Bovine Tuberculosis. OIE Terrestrial Manual 2018.

[20] Goodchild A.V., Downs S.H., Upton P., Wood J.L.N., de la Rua-Domenech R. (2015). Specificity of the comparative skin test for bovine tuberculosis in Great Britain. Vet. Rec..

[21] Karolemeas K., de la Rua-Domenech R., Cooper R., Goodchild A.V., Clifton-Hadley R.S., Conlan A.J.K., Mitchell A.P., Hewinson R.G., Donnelly C.A., Wood J.L.N. (2012). Estimation of the relative sensitivity of the comparative tuberculin skin test in tuberculous cattle herds subjected to depopulation. PLoS ONE.

[22] Petrie A., Watson P. (2013). Statistics for Veterinary and Animal Science.

[23] Djellata N., Yahimi A., Hanzen C., Saegerman C. (2020). Survey of the prevalence of bovine abortions and notification and management practices by veterinary practitioners in Algeria. Rev. Sci. Et Tech. (Int. Off. Epizoot.).

[24] QGIS.org (2026). QGIS Geographic Information System. QGIS Association. http://www.qgis.org.

[25] Hamid M.A., Husain S.M.I., Khan M.K.I., Islam M.N., Biswas M.A.A. (2000). Skin Thickness in Relation to Milk Production of Crossbred CowsPakistan. J. Biol. Sci..

[26] Dowling D. (1995). The thickness of cattle skin. Aust. J. Agric. Res..

[27] Almaw G., Conlan A.J.K., Ameni G., Gumi B., Alemu A., Guta S., Gebre S., Olani A., Garoma A., Shegu D. (2021). The variable prevalence of bovine tuberculosis among dairy herds in Central Ethiopia provides opportunities for targeted intervention. PLoS ONE.

[28] Hiernaux P., Adamou K., Zezza A., Ayantunde A.A., Federighi G. (2016). Milk offtake of cows in smallholder farms of semiarid Sahel: Low yields with high value!. Rev. D’élevage Méd. Vét. Pays Trop..

[29] Harvey N.J., Madden J.M., Casey-Bryars M., Gormley E. (2026). The association between age and bovine tuberculosis diagnosis, using the interferon-gamma (IFN-γ) assay or post-mortem examination in high-risk Irish cattle herds: A retrospective cohort study. Prev. Vet. Med..

[30] Humblet M.F., Boschiroli M.L., Saegerman C. (2009). Classification of worldwide bovine tuberculosis risk factors in cattle: A stratified approach. Vet. Res..

[31] Inangolet F.O., Demelash B., Oloya J., Opuda-Asibo J., Skjerve E. (2008). A cross-sectional study of bovine tuberculosis in the transhumant and agro-pastoral cattle herds in the borde rareas of Katakwi and Moroto districts, Uganda. Trop. Anim. Health Prod..

[32] Ameni G., Aseffa A., Engers H., Young D., Gordon S., Hewinson G., Vordermeier M. (2009). A comparative study on the epidemiology and immuno-pathology of bovine tuberculosis in *Bos indicus* and *Bos taurus* cattle in Ethiopia. Ethiop. J. Health Dev..

[33] Vordermeier M., Ameni G., Berg S., Bishop R., Robertson B.D., Aseffa A., Hewinson R.G., Young D.B. (2012). The influence of cattle breed on susceptibility to bovine tuberculosis in Ethiopia. Comp. Immunol. Microbiol. Infect. Dis..

[34] Elias K., Hussein D., Asseged B., Wondwossen T., Gebeyehu M. (2008). Status of bovine tuberculosis in Addis Ababa dairy farms. Rev. Sci. Tech..

[35] Mehmedi B., Iatrou A.M., Yildiz R., Lamont K., da Costa M.R., De Nardi M., Allepuz A., Niine T., Niemi J.K., Saegerman C. (2025). Economic Perspectives on Farm Biosecurity: Stakeholder Challenges and Livestock Species Considerations. Agriculture.

[36] Tschopp R., Zinsstag J., Conlan A., Gemechu G., Wood J., the ETHICOBOTS consortium (2022). Productivity loss and cost of bovine tuberculosis for the dairy livestock sector in Ethiopia. Prev. Vet. Med..

[37] Souley Kouato B., Elliot F.M., King D.P., Hyera J., Knowles N.J., Ludi A.B., Mioulet V., Matlho G., De Clercq K., Thys E. (2018). Outbreak investigations and molecular characterization of foot-and-mouth disease viruses circulating in south-west Niger. Transbound. Emerg. Dis..

